# Diaqua­bis­{2-hy­droxy-5-[(pyridin-2-yl)methyl­idene­amino]­benzoato-κ^2^ *N*,*N*′}zinc(II) dihydrate

**DOI:** 10.1107/S160053681004496X

**Published:** 2010-11-06

**Authors:** Yue Bing, Xing Li, Meiqin Zha, Yue Lu

**Affiliations:** a Faculty of Materials Science and Chemical Engineering, Ningbo University, Ningbo, Zhejiang 315211, People’s Republic of China

## Abstract

The complex mol­ecule of the title compound, [Zn(C_13_H_9_N_2_O_3_)_2_(H_2_O)_2_]·2H_2_O, has 2 symmetry with the Zn^II^ cation located on a twofold rotation axis. The Zn cation is *N*,*N*′-chelated by two 5-[(pyridin-2-yl)methyl­idene­amino]-2-hy­droxy­benzoate anions and coordinated by two water mol­ecules in a distorted octa­hedral geometry. Within the anionic ligand, the pyridine ring is oriented at a dihedral angle of 49.54 (10)° with respect to the benzene ring. The carboxyl­ate group of the anionic ligand is not involved in coordination but is O—H⋯O hydrogen bonded to the coordinated and uncoordinated water mol­ecules. Weak inter­molecular C—H⋯O hydrogen bonding is also present in the crystal structure.

## Related literature

The title compound is a Schiff base complex; for potential applications of Schiff base compounds, see: Bourque *et al.* (2005 ▶); Donald & Osit (2010 ▶); Feng *et al.* (2007 ▶); Gang *et al.* (2007 ▶); Shanta *et al.* (2003 ▶).
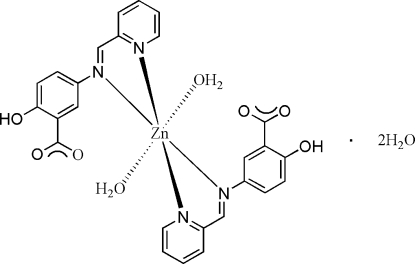


## Experimental

### 

#### Crystal data

[Zn(C_13_H_9_N_2_O_3_)_2_(H_2_O)_2_]·2H_2_O
*M* *_r_* = 619.90
Orthorhombic, 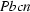

*a* = 15.812 (2) Å
*b* = 10.6962 (15) Å
*c* = 15.636 (2) Å
*V* = 2644.5 (6) Å^3^
*Z* = 4
Mo *K*α radiation
μ = 1.00 mm^−1^
*T* = 296 K
0.43 × 0.32 × 0.27 mm

#### Data collection

Bruker SMART APEX CCD area-detector diffractometer
Absorption correction: multi-scan (*SADABS*; Bruker, 2001 ▶) *T*_min_ = 0.689, *T*_max_ = 0.764
21797 measured reflections
3041 independent reflections
2291 reflections with *I* > 2σ(*I*)
*R*_int_ = 0.038

#### Refinement

*R*[*F* ^2^ > 2σ(*F* ^2^)] = 0.030
*wR*(*F* ^2^) = 0.083
*S* = 1.03
3041 reflections
206 parameters
H atoms treated by a mixture of independent and constrained refinement
Δρ_max_ = 0.33 e Å^−3^
Δρ_min_ = −0.29 e Å^−3^

### 

Data collection: *SMART* (Bruker, 2001 ▶); cell refinement: *SAINT* (Bruker, 2001 ▶); data reduction: *SAINT*; program(s) used to solve structure: *SHELXTL* (Sheldrick, 2008 ▶); program(s) used to refine structure: *SHELXTL*; molecular graphics: *SHELXTL*; software used to prepare material for publication: *SHELXTL*.

## Supplementary Material

Crystal structure: contains datablocks I, global. DOI: 10.1107/S160053681004496X/xu5074sup1.cif

Structure factors: contains datablocks I. DOI: 10.1107/S160053681004496X/xu5074Isup2.hkl

Additional supplementary materials:  crystallographic information; 3D view; checkCIF report

## Figures and Tables

**Table 1:** Selected bond lengths (Å)

Zn1—O1	2.0471 (15)
Zn1—N1	2.1414 (17)
Zn1—N2	2.2746 (14)

**Table 2:** Hydrogen-bond geometry (Å, °)

*D*—H⋯*A*	*D*—H	H⋯*A*	*D*⋯*A*	*D*—H⋯*A*
O1—H1⋯O4^i^	0.80 (3)	1.83 (3)	2.618 (2)	169 (3)
O1—H2⋯O5	0.84 (3)	1.90 (3)	2.744 (3)	178 (3)
O5—H3⋯O3^ii^	0.86 (3)	1.98 (3)	2.808 (2)	161 (3)
O5—H4⋯O2^iii^	0.83 (3)	2.05 (3)	2.881 (3)	174 (3)
O2—H5⋯O3	0.95 (3)	1.58 (3)	2.473 (2)	156 (3)
C6—H6*A*⋯O2^ii^	0.93	2.57	3.346 (3)	142

Symmetry codes: (i) 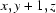; (ii) 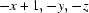; (iii) 
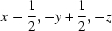
.

**Fig. 1.**
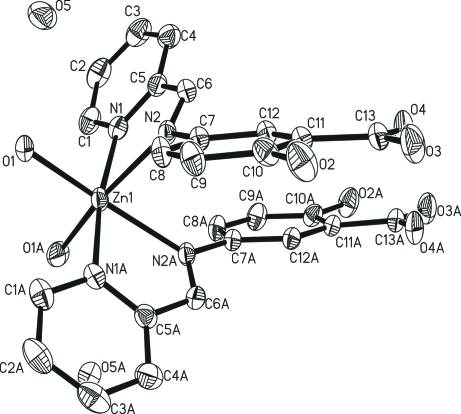
A view of the zinc ion coordination, showing the labeling of the non-H atoms and 30% probability ellipsoids. H atoms have been omitted for clarity.

**Fig. 2.**
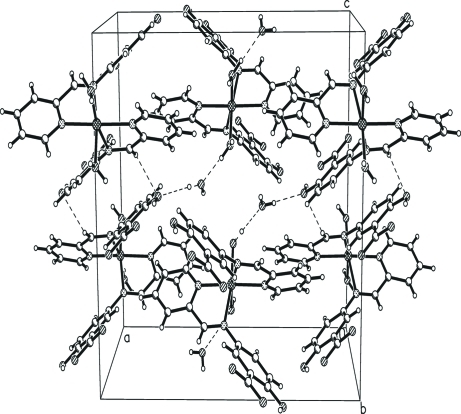
Packing of title complex showing the three dimensional hydrogen bonding network.

## supplementary crystallographic information

### Comment

The current interest in design and synthesis of new Schiff bases and their metal complexes stem from their potential applications in antimicrobial (Bourque *et al.*, 2005), magnetic (Feng *et al.*, 2007; Gang *et al.*, 2007), anticancer (Shanta *et al.*, 2003) and catalytic (Donald *et al.*, 2010). Hereby design and synthesis of new Schiff bases is a important field in coordination chemistry. 5-aminosalicylic acid is widely used in medicine and dye chemistry. 2-pyridinecarboxaldehyde is the intermeadiate of the bisacodyl, which is a popular medicine. We synthesized a new Schiff base 5-((pyridin-2-yl)methyleneamino)-2-hydroxybenzoic acid (C_13_H_9_N_2_O_3_) from 5-aminosalicylic acid and 2-pyridinecarboxaldehyde by nucleophilic addition, followed by a dehydration. The Schiff base can coordinate to the metal atoms through N or O donor atoms. Herein we report the preparation and characterization of the first 5-((pyridin-2-yl)methyleneamino)-2-hydroxybenzoic-zinc(II) complex, [Zn(C_13_H_9_N_2_O_3_)_2_(H_2_O)_2_].2H_2_O.

Single-crystal X-ray diffraction analysis indicates the title complex possesses a mononuclear structure and crystallizes in the orthorhombic, space group *Pbcn* with *Z* = 4. The asymmetric unit consists of two Schiff base ligand, one zinc ion, two coordinated water and two guest water molecule. A view of the zinc ion coordination is shown in Figure 1, where the metal center is coordinated in an octahedral geometry by four N atoms of the Schiff base ligand with Zn—N distances ranging from 2.1416 (17) to 2.2742 (14) Å and two O atoms from water molecules with Zn—O distances ranging from 2.0491 (15) Å. The intromolecular interaction is helpful to the stabilization of the crystal structure (Figure 2).

### Experimental

5-Aminosalicylic acid (1.53 g, 10 mmol), 2-pyridinecarboxaldehyde (1 ml, 10 mmol) and triethylamine (1 ml, 10 mmol) were added to 50 ml e thanol in a round flask, and this mixture was refluxed with agitation for 4 h at 323 K to give an yellow precipitate. After filtration and washing the precipitate with ethanol to give the pure Schiff base 5-((pyridin-2-yl)methyleneamino)-2-hydroxybenzoic acid (2.02 g, 84.00%).

Mixture of 5-((pyridin-2-yl)methyleneamino)-2-hydroxybenzoic acid (0.1 mmol, 0.024 g), Zn(OAc)_2_.2H_2_O (0.1 mmol, 0.022 g) and methanol (20 ml) to give an yellow solution. After evaporating the solution for one week, yellow crystals were obtained (yield, 50%).

### Refinement

H atoms attached to C atoms were placed in calculated positions and treated using a riding-model approximation with C–H = 0.93 Å and U_iso_(H) = 1.2U_eq_(C). H atoms bonded to O atoms were located in a difference Fourier map and refined isotropically.

### Crystal data

**Table:**

[Zn(C_13_H_9_N_2_O_3_)_2_(H_2_O)_2_]·2H_2_O	*F*(000) = 1280
*M**_r_* = 619.90	*D*_x_ = 1.557 Mg m^−^^3^
Orthorhombic, *P**b**c**n*	Mo *K*α radiation, λ = 0.71073 Å
Hall symbol: -P 2n 2ab	Cell parameters from 5445 reflections
*a* = 15.812 (2) Å	θ = 2.3–26.5°
*b* = 10.6962 (15) Å	µ = 1.00 mm^−^^1^
*c* = 15.636 (2) Å	*T* = 296 K
*V* = 2644.5 (6) Å^3^	Block, yellow
*Z* = 4	0.43 × 0.32 × 0.27 mm

### Data collection

**Table:**

Bruker SMART APEX CCD area-detector diffractometer	3041 independent reflections
Radiation source: fine-focus sealed tube	2291 reflections with *I* > 2σ(*I*)
graphite	*R*_int_ = 0.038
φ and ω scans	θ_max_ = 27.5°, θ_min_ = 2.3°
Absorption correction: multi-scan (*SADABS*; Bruker, 2001)	*h* = −20→20
*T*_min_ = 0.689, *T*_max_ = 0.764	*k* = −13→13
21797 measured reflections	*l* = −20→20

### Refinement

**Table:**

Refinement on *F*^2^	Primary atom site location: structure-invariant direct methods
Least-squares matrix: full	Secondary atom site location: difference Fourier map
*R*[*F*^2^ > 2σ(*F*^2^)] = 0.030	Hydrogen site location: inferred from neighbouring sites
*wR*(*F*^2^) = 0.083	H atoms treated by a mixture of independent and constrained refinement
*S* = 1.03	*w* = 1/[σ^2^(*F*_o_^2^) + (0.0389*P*)^2^ + 0.7619*P*] where *P* = (*F*_o_^2^ + 2*F*_c_^2^)/3
3041 reflections	(Δ/σ)_max_ < 0.001
206 parameters	Δρ_max_ = 0.33 e Å^−^^3^
0 restraints	Δρ_min_ = −0.29 e Å^−^^3^

### Special details

**Table:**

Experimental. IR (KBr, cm-1): 3455(*versus*), 3088(w), 2924(w), 2361(w), 1919(w), 1668(*s*), 1602(*m*), 1578(*m*), 1489(*versus*), 1447(*m*), 1364(*versus*), 1299(*m*), 1245(*s*), 1161(*m*), 1084(*m*), 1054(*s*), 959(w), 893(*m*), 839(*s*), 792(*versus*), 655(*m*), 572(*m*), 512(*m*).
Geometry. All e.s.d.'s (except the e.s.d. in the dihedral angle between two l.s. planes) are estimated using the full covariance matrix. The cell e.s.d.'s are taken into account individually in the estimation of e.s.d.'s in distances, angles and torsion angles; correlations between e.s.d.'s in cell parameters are only used when they are defined by crystal symmetry. An approximate (isotropic) treatment of cell e.s.d.'s is used for estimating e.s.d.'s involving l.s. planes.
Refinement. Refinement of *F*^2^ against ALL reflections. The weighted *R*-factor *wR* and goodness of fit *S* are based on *F*^2^, conventional *R*-factors *R* are based on *F*, with *F* set to zero for negative *F*^2^. The threshold expression of *F*^2^ > σ(*F*^2^) is used only for calculating *R*-factors(gt) *etc*. and is not relevant to the choice of reflections for refinement. *R*-factors based on *F*^2^ are statistically about twice as large as those based on *F*, and *R*- factors based on ALL data will be even larger.

### Fractional atomic coordinates and isotropic or equivalent isotropic displacement parameters (Å^2^)

**Table:**

	*x*	*y*	*z*	*U*_iso_*/*U*_eq_	
Zn1	0.5000	0.40959 (2)	0.2500	0.03779 (10)	
O1	0.49550 (12)	0.53276 (14)	0.14990 (11)	0.0590 (4)	
O2	0.70387 (10)	−0.06373 (16)	−0.00402 (11)	0.0623 (4)	
O3	0.62843 (10)	−0.24718 (13)	0.05467 (11)	0.0670 (4)	
O4	0.51579 (10)	−0.22474 (13)	0.13984 (11)	0.0615 (4)	
O5	0.37521 (14)	0.47962 (17)	0.02876 (13)	0.0709 (5)	
N1	0.36496 (10)	0.39448 (14)	0.24957 (9)	0.0414 (4)	
N2	0.47286 (10)	0.24704 (13)	0.16032 (9)	0.0364 (3)	
C1	0.31145 (14)	0.4780 (2)	0.28233 (14)	0.0525 (5)	
H1A	0.3329	0.5440	0.3144	0.063*	
C2	0.22455 (15)	0.4697 (2)	0.27010 (15)	0.0625 (6)	
H2A	0.1889	0.5311	0.2919	0.075*	
C3	0.19182 (15)	0.3703 (2)	0.22564 (16)	0.0643 (6)	
H3A	0.1337	0.3615	0.2190	0.077*	
C4	0.24669 (13)	0.2838 (2)	0.19101 (14)	0.0537 (5)	
H4A	0.2262	0.2158	0.1603	0.064*	
C5	0.33299 (12)	0.30015 (17)	0.20284 (12)	0.0416 (4)	
C6	0.39452 (12)	0.21815 (16)	0.16127 (12)	0.0415 (4)	
H6A	0.3765	0.1445	0.1354	0.050*	
C7	0.53077 (12)	0.16496 (16)	0.11906 (11)	0.0363 (4)	
C8	0.59723 (12)	0.21657 (17)	0.07233 (12)	0.0458 (4)	
H8A	0.6034	0.3029	0.0695	0.055*	
C9	0.65391 (13)	0.14022 (19)	0.03025 (13)	0.0507 (5)	
H9A	0.6977	0.1753	−0.0014	0.061*	
C10	0.64583 (12)	0.01035 (18)	0.03502 (13)	0.0444 (4)	
C11	0.57989 (11)	−0.04244 (16)	0.08294 (12)	0.0386 (4)	
C12	0.52303 (11)	0.03585 (16)	0.12417 (11)	0.0374 (4)	
H12A	0.4790	0.0013	0.1558	0.045*	
C13	0.57306 (13)	−0.18215 (17)	0.09375 (13)	0.0470 (5)	
H1	0.5058 (16)	0.606 (3)	0.152 (2)	0.081 (10)*	
H2	0.4595 (18)	0.517 (3)	0.1115 (18)	0.083 (9)*	
H3	0.3812 (19)	0.417 (3)	−0.005 (2)	0.084 (9)*	
H4	0.326 (2)	0.507 (3)	0.025 (2)	0.124 (14)*	
H5	0.685 (2)	−0.146 (3)	0.009 (2)	0.117 (11)*	

### Atomic displacement parameters (Å^2^)

**Table:**

	*U*^11^	*U*^22^	*U*^33^	*U*^12^	*U*^13^	*U*^23^
Zn1	0.04294 (17)	0.02329 (15)	0.04715 (18)	0.000	−0.00043 (13)	0.000
O1	0.0828 (11)	0.0317 (8)	0.0625 (9)	−0.0157 (8)	−0.0203 (9)	0.0127 (7)
O2	0.0562 (9)	0.0523 (9)	0.0785 (11)	−0.0026 (7)	0.0186 (8)	−0.0231 (8)
O3	0.0642 (9)	0.0356 (8)	0.1011 (12)	0.0057 (7)	0.0064 (9)	−0.0178 (8)
O4	0.0744 (11)	0.0280 (7)	0.0820 (11)	−0.0052 (7)	0.0102 (8)	0.0028 (7)
O5	0.0746 (12)	0.0529 (10)	0.0851 (12)	0.0051 (9)	−0.0225 (10)	−0.0171 (9)
N1	0.0442 (8)	0.0357 (8)	0.0443 (8)	0.0064 (6)	0.0028 (7)	0.0041 (7)
N2	0.0446 (8)	0.0241 (7)	0.0406 (8)	0.0009 (6)	0.0000 (6)	0.0006 (6)
C1	0.0608 (13)	0.0453 (12)	0.0514 (11)	0.0133 (10)	0.0115 (10)	0.0046 (9)
C2	0.0562 (13)	0.0630 (15)	0.0684 (15)	0.0221 (11)	0.0210 (11)	0.0172 (12)
C3	0.0452 (12)	0.0712 (16)	0.0764 (15)	0.0103 (11)	0.0067 (11)	0.0232 (13)
C4	0.0462 (11)	0.0533 (12)	0.0616 (13)	−0.0023 (9)	−0.0050 (10)	0.0108 (10)
C5	0.0427 (10)	0.0356 (9)	0.0466 (10)	0.0005 (8)	−0.0012 (8)	0.0081 (8)
C6	0.0466 (11)	0.0302 (9)	0.0477 (10)	−0.0031 (8)	−0.0059 (8)	−0.0008 (8)
C7	0.0402 (9)	0.0283 (9)	0.0404 (9)	−0.0013 (7)	−0.0009 (8)	−0.0014 (7)
C8	0.0593 (12)	0.0284 (9)	0.0497 (10)	−0.0078 (8)	0.0067 (9)	−0.0001 (8)
C9	0.0572 (12)	0.0423 (11)	0.0525 (11)	−0.0125 (9)	0.0166 (10)	−0.0025 (9)
C10	0.0457 (10)	0.0416 (10)	0.0459 (10)	−0.0015 (8)	0.0015 (9)	−0.0118 (8)
C11	0.0429 (10)	0.0285 (8)	0.0443 (10)	−0.0031 (7)	−0.0054 (8)	−0.0039 (7)
C12	0.0394 (9)	0.0290 (9)	0.0438 (10)	−0.0047 (7)	−0.0017 (7)	0.0000 (7)
C13	0.0533 (12)	0.0284 (9)	0.0593 (12)	0.0014 (8)	−0.0092 (10)	−0.0067 (9)

### Geometric parameters (Å, °)

**Table:**

Zn1—O1^i^	2.0471 (15)	C1—H1A	0.9300
Zn1—O1	2.0471 (15)	C2—C3	1.372 (4)
Zn1—N1^i^	2.1414 (17)	C2—H2A	0.9300
Zn1—N1	2.1414 (17)	C3—C4	1.379 (3)
Zn1—N2	2.2746 (14)	C3—H3A	0.9300
Zn1—N2^i^	2.2746 (14)	C4—C5	1.388 (3)
O1—H1	0.81 (3)	C4—H4A	0.9300
O1—H2	0.84 (3)	C5—C6	1.462 (3)
O2—C10	1.357 (2)	C6—H6A	0.9300
O2—H5	0.95 (3)	C7—C12	1.389 (2)
O3—C13	1.274 (2)	C7—C8	1.394 (3)
O4—C13	1.244 (2)	C8—C9	1.380 (3)
O5—H3	0.86 (3)	C8—H8A	0.9300
O5—H4	0.84 (4)	C9—C10	1.397 (3)
N1—C1	1.333 (2)	C9—H9A	0.9300
N1—C5	1.344 (2)	C10—C11	1.403 (3)
N2—C6	1.277 (2)	C11—C12	1.388 (2)
N2—C7	1.423 (2)	C11—C13	1.508 (2)
C1—C2	1.390 (3)	C12—H12A	0.9300
			
O1^i^—Zn1—O1	99.88 (10)	C2—C3—H3A	120.6
O1^i^—Zn1—N1^i^	90.66 (6)	C4—C3—H3A	120.6
O1—Zn1—N1^i^	94.92 (7)	C3—C4—C5	118.8 (2)
O1^i^—Zn1—N1	94.92 (7)	C3—C4—H4A	120.6
O1—Zn1—N1	90.66 (6)	C5—C4—H4A	120.6
N1^i^—Zn1—N1	171.34 (8)	N1—C5—C4	122.45 (18)
O1^i^—Zn1—N2	165.91 (6)	N1—C5—C6	116.20 (16)
O1—Zn1—N2	90.80 (6)	C4—C5—C6	121.29 (18)
N1^i^—Zn1—N2	97.61 (5)	N2—C6—C5	120.37 (16)
N1—Zn1—N2	75.65 (6)	N2—C6—H6A	119.8
O1^i^—Zn1—N2^i^	90.80 (6)	C5—C6—H6A	119.8
O1—Zn1—N2^i^	165.91 (6)	C12—C7—C8	119.37 (17)
N1^i^—Zn1—N2^i^	75.65 (6)	C12—C7—N2	122.05 (16)
N1—Zn1—N2^i^	97.61 (5)	C8—C7—N2	118.58 (15)
N2—Zn1—N2^i^	80.29 (7)	C9—C8—C7	120.36 (17)
Zn1—O1—H1	126 (2)	C9—C8—H8A	119.8
Zn1—O1—H2	115.9 (19)	C7—C8—H8A	119.8
H1—O1—H2	111 (3)	C8—C9—C10	120.25 (17)
C10—O2—H5	103 (2)	C8—C9—H9A	119.9
H3—O5—H4	109 (3)	C10—C9—H9A	119.9
C1—N1—C5	118.25 (18)	O2—C10—C9	119.64 (18)
C1—N1—Zn1	125.53 (15)	O2—C10—C11	120.52 (18)
C5—N1—Zn1	115.68 (12)	C9—C10—C11	119.79 (17)
C6—N2—C7	118.70 (15)	C12—C11—C10	119.14 (16)
C6—N2—Zn1	111.15 (12)	C12—C11—C13	119.98 (17)
C7—N2—Zn1	129.02 (12)	C10—C11—C13	120.80 (17)
N1—C1—C2	122.1 (2)	C11—C12—C7	121.09 (17)
N1—C1—H1A	118.9	C11—C12—H12A	119.5
C2—C1—H1A	118.9	C7—C12—H12A	119.5
C3—C2—C1	119.5 (2)	O4—C13—O3	125.32 (18)
C3—C2—H2A	120.3	O4—C13—C11	118.69 (17)
C1—C2—H2A	120.3	O3—C13—C11	115.98 (18)
C2—C3—C4	118.8 (2)		

Symmetry codes: (i) −*x*+1, *y*, −*z*+1/2.

### Hydrogen-bond geometry (Å, °)

**Table:**

*D*—H···*A*	*D*—H	H···*A*	*D*···*A*	*D*—H···*A*
O1—H1···O4^ii^	0.80 (3)	1.83 (3)	2.618 (2)	169 (3)
O1—H2···O5	0.84 (3)	1.90 (3)	2.744 (3)	178 (3)
O5—H3···O3^iii^	0.86 (3)	1.98 (3)	2.808 (2)	161 (3)
O5—H4···O2^iv^	0.83 (3)	2.05 (3)	2.881 (3)	174 (3)
O2—H5···O3	0.95 (3)	1.58 (3)	2.473 (2)	156 (3)
C6—H6A···O2^iii^	0.93	2.57	3.346 (3)	142

Symmetry codes: (ii) *x*, *y*+1, *z*; (iii) −*x*+1, −*y*, −*z*; (iv) *x*−1/2, −*y*+1/2, −*z*.
